# Association between support of basic psychological needs and stress response as mediated by motivation for solitude

**DOI:** 10.1371/journal.pone.0304846

**Published:** 2024-06-21

**Authors:** Gen Takagi, Michiaki Shibata, Yumi Nakagawa

**Affiliations:** 1 Faculty of Comprehensive Welfare, Tohoku Fukushi University, Sendai, Japan; 2 Faculty of Business Administration, Hokkai-Gakuen University, Sapporo, Japan; University of Campinas - UNICAMP, BRAZIL

## Abstract

This study examined the relationship between not self-determined / self-determined solitude and stress responses, as well as the effect of supporting / thwarting of basic psychological needs. The sample consisted of 606 Japanese people aged 20 years and older. We used a cross-sectional survey to measure their motivations for solitude, supporting / thwarting of basic psychological needs, and stress responses. Not self-determined solitude was negatively correlated with supporting of basic psychological needs and positively correlated with thwarting of basic psychological needs. In addition, a cutoff score of 16 for not self-determined solitude was found to be optimal for identifying individuals with a high stress response. Mediation analysis revealed that supporting / thwarting of basic psychological needs has direct effects on stress responses and indirect effects mediated by not self-determined solitude. These findings suggest that fostering environments that support basic psychological needs of autonomy, competence, and relatedness can alleviate not self-determined solitude and reduce stress responses.

## Introduction

Loneliness has been shown to be associated with poor physical and mental health and with unhealthy lifestyle behaviors such as smoking, low physical activity, and low fruit and vegetable consumption [1]. For instance, loneliness was found to be correlated with social anxiety [2] and depression [3] and was shown to interfere with the development of identity [4]. However, comprehensive analyses of the effects of loneliness must distinguish between wanted and unwanted loneliness.

### Motivation for solitude

The concept of motivation for solitude is useful in distinguishing between wanted and unwanted loneliness. Nicol [5] conceptualized loneliness based on the self-determination theory (SDT), which distinguishes between self-determined and not self-determined solitude. Self-determined solitude implies being alone for positive reasons, such as meditation, creativity, and self-reflection, whereas not self-determined solitude implies being alone for negative reasons, such as social anxiety, peer rejection, and lack of friendship [6]. Not self-determined solitude is consistent with the concept of unwanted loneliness and has been shown to be positively correlated with loneliness and social anxiety [5]. Moreover, higher levels of not self-determined solitude are associated with higher levels of anxiety, depression, hopelessness, and loneliness and lower levels of social connectedness [7].

In contrast, self-determined solitude has not been negatively associated with loneliness or social anxiety [5]. Self-determined solitude is associated with higher happiness, desire for autonomy, and fulfillment [6, 8, 9]. In addition, self-determined solitude may lead to relaxation and stress reduction [10]. Thus, solitude can have different effects depending on whether it is self-determined. The concept of motivation for solitude allows a more precise examination of its various effects. Therefore, distinguishing between self-determined and not self-determined solitude is important when examining unwanted loneliness.

### Self-determination theory

According to the SDT, three fundamental psychological requirements must be satisfied to promote self-determined behavior: need for autonomy, need for competence, and need for relatedness [11]. Research shows positive correlations between the fulfillment of these basic psychological needs and well-being [12–14]. Furthermore, supportive behaviors that facilitate the fulfillment of these needs have been linked to increased happiness and decreased depression [15].

From the perspective of the SDT, self-determination toward solitude is thought to increase when the three basic psychological needs are supported, and conversely, self-determination toward solitude is thought to decrease when the three basic psychological needs are thwarted. Consequently, bolstering basic psychological needs fulfillment is predicted to enhance individual self-determination in relation to solitude, thereby reducing the experience of not self-determined solitude. However, to date, no empirical research has investigated the relationship between support of basic psychological needs and not self-determined solitude. Therefore, this study examined the effects of need-supportive and need-thwarting behaviors on self-determined and not self-determined solitude.

### Effects of not self-determined and self-determined solitude on stress response

Studies on the negative effects of not self-determined solitude have focused primarily on anxiety, depression, and hopelessness [5, 7]. However, depression and anxiety are merely aspects of mental health, whereas stress response is a more comprehensive concept. Suzuki et al. [16] identified “irritability and anger” and “helplessness” as aspects of the stress response in addition to “depression and anxiety,” and developed a scale to comprehensively measure them. This Stress Response Scale (SRS) is a comprehensive measure of mental health that includes depression and anxiety, among other aspects. The overall negative impact of not self-determined solitude can be examined using such a measure of stress response as a comprehensive indicator of mental health.

Furthermore, the scale of stress responses can be utilized by categorizing scores into “low,” “normal,” “somewhat high,” and “high,” to assess the extent to which high levels of solitude are associated with a high stress response. It is therefore also possible to investigate the cut-off point for not self-determined solitude at which serious stress responses may occur. The negative effects of not self-determined solitude have been highlighted in several studies, but it has not been indicated at what point it becomes problematic. Therefore, the present study investigates the cut-off point for not self-determined solitude to determine a high stress response.

Moreover, as noted above, self-determined solitude may lead to relaxation and stress reduction [10]. However, a study of Japanese subjects by Emura and Miyazaki [9] reported a weak correlation (0.08) between self-determined solitude and loneliness. This may be due to the difference that Nguyen et al. [10] conducted their experiment with participants of diverse ethnicities, while Emura and Miyazaki [9] conducted a cross-sectional study with Japanese subjects. Since the present study is a cross-sectional survey of Japanese subjects, it can be assumed that the negative aspects of self-determined solitude will be shown in the same way as Emura and Miyazaki [9]. To explore this point, this study also examined the effects of self-determined solitude on stress responses.

Thus, this study’s primary objective was to investigate the relationship between not self-determined / self-determined solitude and stress responses and determine the extent to which high levels of the former are associated with the latter. Additionally, while previous research has suggested that support of basic psychological needs may affect not self-determined / self-determined solitude, this association has not yet been explored. Therefore, the second objective of this study was to investigate the association between basic psychological needs and not self-determined / self-determined solitude. Furthermore, the third objective of this study was to examine the influence of basic psychological needs on stress responses through the mediation of not self-determined / self-determined solitude.

## Materials and methods

### Procedure

A web-based survey was conducted from June 8 and June 22, 2022, with a sample of Japanese people aged 20 years and older. Survey participants were recruited through CrowdWorks, a Japanese company specializing in crowdsourcing services (https://crowdworks.jp/). A wide variety of individuals are registered to participate in simple tasks on CrowdWorks. We posted our survey request on CrowdWorks and invited individuals to participate. We offered participants a monetary incentive of ¥50.

The content of the study and questionnaire were explained on the first screen. Participants were informed that responding to the questions was voluntary, that their responses would be statistically analyzed, that they could withdraw at any time, that the survey would be conducted anonymously, and that no personal information would be disclosed. Informed consent was obtained online through the survey platform. Participants were allowed to proceed to the survey response page only after providing informed consent. This study was approved by the Research Ethics Review Committee at the first author’s institution.

A total of 606 people (229 men, 377 women; mean age = 39.54 years, SD = 10.07; age range = 21–76 years) participated in the survey.

### Questionnaire

To measure the variables, the survey comprised a sociodemographic questionnaire and three psychological measurement scales. Participants who agreed to be involved in the study completed each section as described below.

#### Demographic characteristics

Participants were asked to provide their gender, age, and job status.

#### Basic psychological needs supporting / thwarting

To measure the supporting and thwarting of basic psychological needs, we used the Japanese version of the Interpersonal Behaviours Questionnaire adapted for a Japanese population by Xiao and Toyama [15] based on the scale originally developed by Rocchi et al. [17]. This scale measures perceptions of the behaviors of others that either support or thwart basic psychological needs. The scale comprises six subfactors, each with four items: autonomy-supportive, autonomy-thwarting, competence-supportive, competence-thwarting, relatedness-supportive, and relatedness-thwarting behaviors. Xiao and Toyama [15] confirmed the reliability and validity of this scale. Items are scored on a seven-point Likert scale ranging from 1 (*not at all agree*) to 7 (*completely agree*). The total score for each subfactor is calculated and used as an indicator of supported or thwarted basic psychological needs. In this study, Cronbach’s alphas were .886 for autonomy-supportive, .895 for autonomy-thwarting, .799 for Competence-supportive, .739 for competence-thwarting, .853 for Relatedness-supportive, and .832 for relatedness-thwarting behaviors, indicating sufficient reliability.

#### Motivation for solitude

To measure motivation for solitude, we used the Japanese version of the Motivation for Solitude Scale–Short Form (MSS-SF), which was originally developed by Thomas and Azmitia [6], and adapted for use in Japanese populations by Emura and Miyazaki [9]. This scale measures an individual’s motivation for solitude and comprises two subfactors: self-determined solitude (8 items) and not self-determined solitude (6 items). Emura and Miyazaki [9] confirmed the scale’s reliability and validity. Items are scored on a four-point Likert scale ranging from 1 (*not at all important*) to 4 (*very important*). The total score for each subfactor is calculated and used as an indicator of motivation for solitude. In this study, Cronbach’s alphas were .782 for self-determined solitude and .893 for not self-determined solitude, indicating sufficient reliability.

#### Stress response

To measure the stress response, we used the Stress Response Scale-18 developed by Suzuki et al. [16]. This scale measures psychological stress responses to stressful situations experienced in daily life. It comprises three subfactors, each with six items: depression-anxiety, irritability-anger, and helplessness. Suzuki et al. [16] confirmed the reliability and validity of this scale. Items are scored on a four-point Likert scale ranging from 0 (*completely disagree*) to 3 (*Strongly Agree*). Total scores are calculated for all items as an indicator of stress response. In this study, Cronbach’s alpha was .942.

### Data analysis

SPSS (version 27.0) was used for data analysis. The scale’s reliability was assessed using the Cronbach’s alpha coefficient. Pearson correlation coefficients were calculated to analyze the correlations. A two-tailed test was employed in all statistical analyses. A p-value of less than 0.05 was considered indicative of a significant difference in all statistical analyses. Receiver operating characteristic (ROC) curve analysis is a statistical technique that enables the identification of optimal cutoff points that correspond to the best possible combination of sensitivity and specificity for a given screening tool. To determine the diagnostic accuracy of the ROC curve, the nonparametric statistical area under the curve (AUC) was used. According to Swets et al. [18], AUC values ≥ 0.90 are “excellent,” ≥ 0.80 “good,” ≥ 0.70 “fair,” and < 0.70 “poor.” The optimal cutoff points for the problematic isolation, using the “MSS-SF,” were determined based on the Youden index [19].

## Results

### Descriptive statistics

The total scores for all scales were calculated and used in the analysis. Table 1 shows the descriptive statistics for each scale.

**Table 1:** Descriptive statistics for each scale.

Variables	*M*	*SD*	*Min*	*Max*
Basic psychological needs supporting / thwarting				
Autonomy-supportive behaviors	19.06	4.00	4.00	28.00
Autonomy-thwarting behaviors	12.66	4.95	4.00	28.00
Competence-supportive behaviors	17.49	4.04	4.00	28.00
Competence-thwarting behaviors	13.17	3.88	4.00	28.00
Relatedness-supportive behaviors	17.68	4.26	4.00	28.00
Relatedness-thwarting behaviors	12.67	4.54	4.00	28.00
Motivation for solitude				
Self-determined solitude	24.35	3.70	14.00	32.00
Not self-determined solitude	14.37	4.04	6.00	24.00
Stress response	17.01	12.13	0.00	53.00
Depression-anxiety	5.75	4.72	0.00	18.00
Irritability-anger	4.60	4.28	0.00	18.00
Helplessness	6.65	4.53	0.00	18.00

The datasets generated and analyzed during the current study are available online at https://osf.io/zy2hx/?view_only=c529b62ace2948e98509f1c187ea8c57.

### Relationship between the motivation for solitude and basic psychological need supporting / thwarting

A correlation analysis was performed to examine the relationship between motivation for solitude and basic psychological needs supporting / thwarting (Table 2). Self-determined solitude was positively correlated with autonomy-thwarting, competence-supportive, and competence-thwarting behaviors. Not self-determined solitude was negatively correlated with all thwarting behaviors and positively correlated with all supportive behaviors.

**Table 2:** Relationship between the motivation for solitude and basic psychological needs supporting / thwarting.

	Self-determined solitude	Not self-determined solitude
Autonomy-supportive behaviors	.06	-.42^***^
Autonomy-thwarting behaviors	.10^*^	.37^***^
Competence-supportive behaviors	.13^**^	-.39^***^
Competence-thwarting behaviors	.10^*^	.35^***^
Relatedness-supportive behaviors	.07	-.51^***^
Relatedness-thwarting behaviors	.03	.50^***^

^*^p < .05

^**^p < .01

^***^p < .001

### Relationship between the motivation for solitude and stress response

A correlation analysis was performed to examine the relationship between motivation for solitude and stress response (Table 3). Self-determined solitude was positively correlated with overall stress response and helplessness. Not self-determined solitude was negatively correlated with all aspects of stress response.

**Table 3:** Relationship between the motivation for solitude and stress response.

	Self-determined solitude	Not self-determined solitude
Stress response	.09^*^	.51^***^
Depression-anxiety	.07	.45^***^
Irritability-anger	.06	.38^***^
Helplessness	.11^**^	.53^***^

^*^p < .05

^**^p < .01

^***^p < .001

### ROC curve analyses

**Fig 1:**
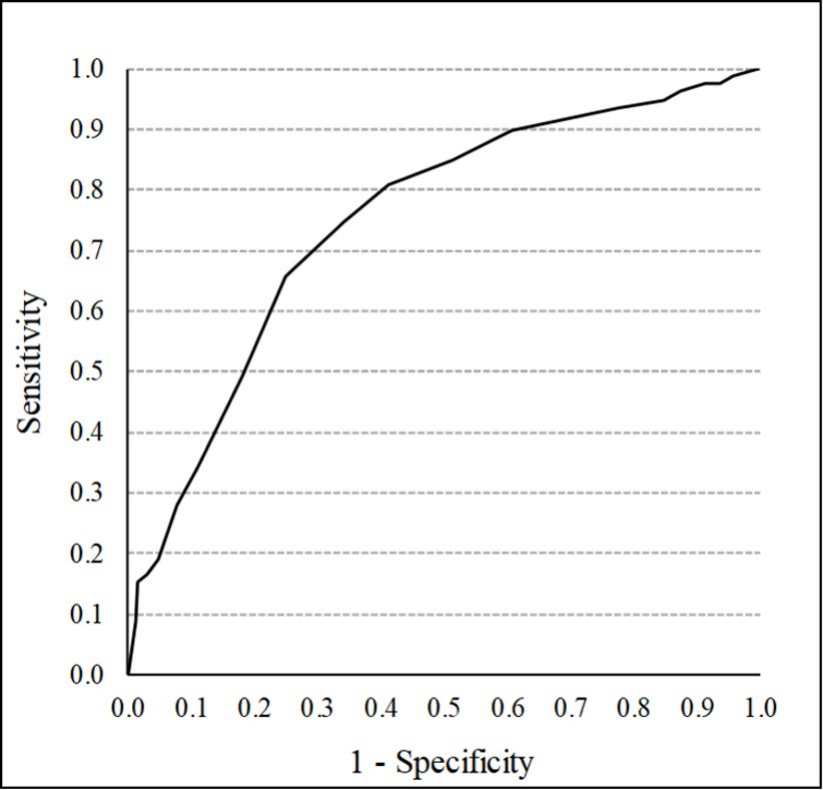
Positive and false positive rates for ROC analysis.

The Stress Response Scale-18 considered scores of 31 or higher for men and 32 or higher for women to indicate a high stress response. Therefore, in line with these reference values, participants were classified into a positive group with a high stress response (29 male and 50 female) and a negative group without a high stress response (200 male and 327 female). Cutoff values for the motivation for isolation were examined with this category. Among the motivations for solitude, self-determined solitude was only weakly related to stress response. If the association is weak, the ROC curve analysis is likely to fail to discriminate adequately. Therefore, this study analyzed only the cutoff values for not self-determined solitude. The ROC curve analysis showed an AUC of .749, indicating fair discriminative accuracy (Fig 1). The optimal cutoff value was determined based on the Youden index, and a score of 16 points (sensitivity = .658, specificity = .751) was shown to be the optimal cutoff value [19].

### Direct and indirect effects of basic psychological needs supporting / thwarting on stress response mediated by motivation for solitude

To examine the direct and indirect effects of basic psychological needs supporting / thwarting on stress response as mediated by motivation for solitude, the bootstrap method (bootstrap sample size: 5000) was used with PROCESS v4.0 created by Hayes [20]. According to Shrout and Bolger [21], an indirect effect is considered significant when the 95% confidence interval obtained using the bootstrap method does not contain zero. The results showed that all subfactors of basic psychological needs supporting / thwarting had a direct effect on stress response and an indirect effect mediated by not self-determined solitude (Table 4). Specifically, basic psychological needs support showed positive direct and indirect effects, whereas thwarted basic psychological needs showed negative direct and indirect effects.

**Table 4:** The direct and indirect effects of supporting / thwarting of basic psychological needs on stress response.

Independent variable	Mediating variable	Dependent variable	Effect size	*SE*	95% CI	
Autonomy-supportive behaviors	= = = = = >	Stress response	-.53	.12	-.76	-.30
	= > Self-determined solitude = >		.00	.00	-.01	.03
	= > Not self-determined solitude = >		-.55	.07	-.69	-.42
Autonomy-thwarting behaviors	= = = = = >	Stress response	.55	.09	.37	.73
	= > Self-determined solitude = >		.00	.01	-.02	.02
	= > Not self-determined solitude = >		.38	.05	.29	.49
Competence-supportive behaviors	= = = = = >	Stress response	-.30	.12	-.53	-.07
	= > Self-determined solitude = >		.01	.02	-.02	.04
	= > Not self-determined solitude = >		-.54	.07	-.69	-.41
Competence-thwarting behaviors	= = = = = >	Stress response	.76	.11	.53	.98
	= > Self-determined solitude = >		.00	.01	-.03	.02
	= > Not self-determined solitude = >		.46	.07	.33	.59
Relatedness-supportive behaviors	= = = = = >	Stress response	-.54	.12	-.77	-.31
	= > Self-determined solitude = >		.01	.01	-.01	.03
	= > Not self-determined solitude = >		-.59	.07	-.73	-.46
Relatedness-thwarting behaviors	= = = = = >	Stress response	.51	.11	.30	.72
	= > Self-determined solitude = >		.00	.01	-.01	.02
	= > Not self-determined solitude = >		.55	.06	.43	.67

## Discussion

This study’s findings indicate that self-determined solitude is positively correlated with autonomy-thwarting, competence-supportive, and competence-thwarting behaviors. Conversely, not self-determined solitude was negatively correlated with autonomy-supportive behaviors, competence-supportive behaviors, and relatedness-supportive behaviors, and showed positive correlations with autonomy-thwarting behaviors, competence-thwarting behaviors, and relatedness-thwarting behaviors. Furthermore, self-determined solitude was positively correlated with total SRS scores and helplessness, whereas not self-determined solitude showed a positive correlation with all aspects of stress response.

ROC analysis revealed that the optimal cutoff score for not self-determined solitude was 16 points for discriminating high-stress individuals. The results of the mediation analysis suggest that behaviors that support autonomy, competence, and relatedness in individuals mediate not self-determined solitude and reduce stress responses. Conversely, behaviors that thwart autonomy, competence, and relatedness had a mediating effect on not self-determined solitude and exacerbated stress responses. These findings are discussed below.

### Self-determined solitude and basic psychological needs supporting / thwarting and stress responses

This study’s results indicate that those who perceived behaviors from their surroundings that thwart autonomy, support competence, and thwart competence had higher levels of self-determined solitude. Specifically, a positive correlation was observed between competence and self-determined solitude for both supportive and thwarting behaviors. This suggests that perceiving either supportive or thwarting behaviors can enhance individuals’ motivation for self-determined solitude.

Competence-supportive behaviors have a positive impact on an individual’s competence, enabling them to enjoy solitude. Competence-thwarting behaviors have a detrimental effect on individuals’ competence, leading to reduced expectations and a preference for solitude over social interaction. Similarly, autonomy-thwarting behaviors may increase the inclination towards self-determined solitude because of a loss of expectations from others.

Xiao and Toyama [15] reported a weak negative correlation of -.16 between supportive and thwarting competence behaviors. However, a moderate negative correlation of -.49 was found between supportive and thwarting behaviors related to autonomy and -.64 between supportive and thwarting behaviors related to relationships. This indicates that for autonomy- and relationship-related behaviors, more supportive behaviors are often associated with fewer thwarting behaviors, whereas for competence-related behaviors, high levels of both supportive and thwarting behaviors may occur. Those who perceive more of both supportive and thwarting competence behaviors in their environment may be exposed to others’ judgments more frequently and show greater concern regarding those evaluations. Consequently, they may actively choose solitude to avoid interpersonal relationships.

If the loss of expectations of one’s environment increases self-determined solitude, relational thwarting would also lead to a loss of expectations and an increase in self-determined solitude. However, no such correlations were observed in the present study. Relatedness-thwarting behaviors were assessed using items such as "They don’t comfort me when I am down." Individuals who experience these behaviors may feel disheartened about their surroundings, while others may have high expectations of receiving comfort from others. Furthermore, those who are exposed to relatedness-thwarting behaviors may struggle with being with others or alone [22]. Thus, the effects that occur when a relationship is thwarted may depend on the characteristics of the recipients and the situation in which they are placed. Consequently, the relationship thwarting did not show an association with self-determined solitude.

Notably, no correlations were found between autonomy-supportive and relatedness-supportive behavior and self-determined solitude. A reasonable assumption would be that individuals who receive a significant amount of autonomy- and relatedness-supportive behaviors from others are generally esteemed by their peers and form close relationships with them. Hence, the presence of such individuals may either promote the enjoyment of solitude or foster an appreciation for spending time with others. Both possibilities are reasonable, and autonomy and relatedness support are not considered the primary determinants of self-determined solitude.

In this study, self-determined solitude and stress response were found to be weakly and positively correlated (.09). Emura and Miyazaki [9] reported a weak correlation (.08) between self-determined solitude and loneliness, which was consistent with this study. However, it is important to note that this association is weak, whereas the association for not self-determined solitude is strong (.59). Therefore, although the negative aspects of self-determined loneliness were less pronounced than the negative aspects of not self-determined solitude, it is reasonable to assume that a small percentage of Japanese with high self-determined solitude include those who experience feelings of helplessness.

Moreover, self-determination is more likely to prevent the negative effects of solitude on stress responses than not self-determination. Moustakas [23] noted that “there are times when he will realize how alone he is as an individual. This is the inevitable loneliness of human existence.” However, if solitude is unavoidable, it would be preferable to spend one’s time in a more favorable mental state by actively seeking out self-determined rather than not self-determined solitude. As noted above, previous studies have demonstrated a correlation between self-determined solitude and higher levels of happiness, need for autonomy, and fulfillment [6, 8, 9]. Consequently, it is crucial to cultivate self-determined solitude to mitigate the potentially detrimental effects of solitude and optimize its beneficial outcomes.

### Not self-determined solitude and basic psychological needs supporting / thwarting and stress responses

The present study revealed a negative correlation between not self-determined solitude and support of basic psychological needs and a positive correlation between not self-determined solitude and thwarting of basic psychological needs. These findings imply that the absence of thwarting of basic psychological needs and support of basic psychological needs can alleviate not self-determined solitude. This study’s findings suggest that the fulfillment of basic psychological needs, namely autonomy, competence, and relatedness, can help reduce not self-determined solitude.

Not self-determined solitude was found to be moderately positively correlated with all aspects of stress response. This aligns with previous research, suggesting that not self-determined solitude can exacerbate social anxiety and depressive symptoms [5, 7]. The results of this study suggest that not self-determined solitude is linked to not only anxiety and depression but also to various aspects of the stress response, including anger, irritability, and helplessness.

The present findings, as indicated by the ROC curve analysis, suggest that a cutoff value of 16 points for not self-determined solitude is optimal for distinguishing individuals exhibiting a heightened stress response. Despite the widely recognized negative consequences of not self-determined solitude [24, 25], no established cutoff values for poor mental health exist. Thus, the cutoff values identified in this study provide a useful framework for future investigations into the individual effects of not self-determined solitude.

The mediation analysis results indicated that support of basic psychological needs had positive direct effects on stress responses and indirect effects through not self-determined solitude. Conversely, thwarted basic psychological needs exhibited negative direct effects on stress responses and indirect effects through not self-determined solitude. Xiao and Toyama [15] also found associations between encouraging the fulfillment of basic psychological needs and both higher well-being and lower depression. These results provide evidence for the notion that less support for or more thwarting of basic psychological needs has a detrimental impact on mental health and that not self-determined solitude functions as a mediating factor in this relationship with stress responses. Hence, reducing not self-determined solitude may be a useful strategy for mitigating the negative repercussions stemming from inadequate support or excessive thwarting of basic psychological needs on stress responses.

### Limitations

This study has some limitations that should be discussed. First, this study is based on the results of a cross-sectional survey. A longitudinal study is needed to establish causality and make stronger claims regarding the influence of basic psychological need-supportive and thwarting behaviors on stress responses through the mediation of not self-determined solitude. Second, the participants were limited to Japanese adults. Additional research is required to determine whether these findings can be generalized to other cultural backgrounds and children. Finally, although this study examined motivations for solitude, it did not consider individuals’ specific situations and contexts regarding solitude. A more in-depth investigation of individual-specific situations and contexts may allow us to propose more practical guidance.

## Conclusions

This study demonstrated the effects of behaviors that support or thwart basic psychological needs on stress responses through the mediation of not self-determined solitude. These findings suggest the importance of increasing behaviors that support basic psychological needs to alleviate not self-determined solitude and reduce stress responses. This is particularly valuable because behaviors that support basic psychological needs can be enhanced by constructing a supportive environment, allowing us to propose actionable supportive interventions to reduce not self-determined solitude and stress responses. Future research should explore the causal dynamics of the issue through a longitudinal study to clarify that behaviors that support or thwart basic needs increase not self-determined solitude and consequently enhance the stress response. Furthermore, effective guidelines for the prevention of problematic loneliness need to be proposed through a comprehensive examination of the influence of cultural contexts and individual circumstances to prevent problems caused by not self-determined solitude.
